# Defects in magnetic domain walls after single-shot all-optical switching

**DOI:** 10.1063/4.0000287

**Published:** 2025-04-18

**Authors:** Daniel Metternich, Kai Litzius, Sebastian Wintz, Kathinka Gerlinger, Sascha Petz, Dieter Engel, Themistoklis Sidiropoulos, Riccardo Battistelli, Felix Steinbach, Markus Weigand, Steffen Wittrock, Clemens von Korff Schmising, Felix Büttner

**Affiliations:** 1 Helmholtz-Zentrum Berlin, 14109 Berlin, Germany; 2 Experimental Physics V, Center for Electronic Correlations and Magnetism, University of Augsburg, 86159 Augsburg, Germany; 3 Max Born Institute for Nonlinear Optics and Short Pulse Spectroscopy, 12489 Berlin, Germany

## Abstract

Helicity-independent all-optical switching (HI-AOS) is the fastest known way to switch the magnetic order parameter. While the switching process of extended areas is well understood, the formation of domain walls enclosing switched areas remains less explored. Here, we study domain walls around all-optically nucleated magnetic domains using x-ray vector spin imaging and observe a high density of vertical Bloch line defects. Surprisingly, the defect density appears to be independent of optical pulse parameters, significantly varies between materials, and is only slightly higher than in domain walls generated by field cycling. A possible explanation is given by time-resolved Kerr microscopy, which reveals that magnetic domains considerably expand after the initial AOS process. During this expansion, and likewise during field cycling, domain walls propagate at speeds above the Walker breakdown. Micromagnetic simulations suggest that at such speeds, domain walls accumulate defects when moving over magnetic pinning sites, explaining similar defect densities after two very different switching processes. The slightly larger defect density after AOS compared to field-induced switching indicates that some defects are created already when the domain wall comes into existence. Our work shows that engineered low-pinning materials are a key ingredient to uncover the intrinsic dynamics of domain wall formation during ultrafast all-optical switching.

## I. INTRODUCTION

Switchable order parameters (e.g., ferroelectric polarization or magnetization) are key ingredients of modern and next-generation information technologies.^1–4^ In this context, magnetization stands out as an order parameter that is both persistent and switchable without degrading the underlying material. The physics of order parameter switching is subject of intense research, for example, to identify fundamental limits of the process in terms of speed, energy efficiency, and reproducibility, where examples of the latter are switching field distributions in magnetic hard disks and switching current distributions in magnetic random access memory (MRAM) cells.^5,6^ In all these research areas, defects play a central role, including defects of intrinsic nature, i.e., domain wall defects in the order parameter itself. Such domain wall defects can be detrimental, for example, by adding stochasticity to the switching dynamics or by affecting the scalability and long-term stability of final-state switched volumes (for example, in skyrmion-based information technologies^7^). However, they can also provide valuable insights into the underlying switching trajectories. It is, therefore, of both fundamental and applied interest to study the appearance of defects in domain walls, especially after ultrafast excitation.

The emergence of defects can be best understood in the framework of phase transitions. For example, field- or current-induced switching of magnetic memory cells is a first-order phase transition, as it exhibits hysteresis. Consequently, this type of switching usually proceeds via nucleation and growth.^8^ Both of these stages of the switching process are influenced by Arrhenius activation and defects, resulting in stochasticity in space and time, i.e., switching distributions as discussed before. On the contrary, second order transitions, such as heat-induced transitions or continuous rotation, may, in principle, proceed homogeneously via transient vanishing of the order parameter. In practice, however, extrinsic defects can still lead to spatial modulations. Moreover, if the transition involves spontaneous symmetry breaking (as, for example, in a domain wall, where clockwise and counterclockwise Bloch configurations are equivalent), then intrinsic defects can emerge even in a perfectly homogeneous material by a mechanism introduced by Kibble and Zurek.^9,10^ In brief, Kibble and Zurek argued that spatially separated areas of the same sample need to select their order parameter orientation independently if their distance is larger than the speed of information times the duration of the phase transition. The result is a defect density that scales inversely with the duration of the phase transition. This Kibble–Zurek mechanism of defect generation during second order phase transitions was verified in magnetic^11^ as well as ferroelectric materials,^12,13^ and it appears to be particularly relevant for ultrafast switching processes.

One of such ultrafast switching processes is single-shot, helicity-independent all-optical switching (HI-AOS) of the magnetic order parameter. HI-AOS is the fastest known way to switch the magnetization;^14,15^ the process happens on a picosecond timescale after femtosecond to low picosecond optical excitation.^16^ HI-AOS has been observed mostly in Gd-containing rare-earth transition-metal ferrimagnets and is well understood in a macrospin picture.^17–20^ In this picture, the transition-metal demagnetizes first. Gd, which is antiferromagnetically coupled to the transition metals, demagnetizes later, partially by transferring angular momentum to the transition-metal atoms.^18^ A real-space dimension to the understanding of HI-AOS was chiefly developed by Graves *et al.*, who showed that the supposedly homogeneous rare-earth transition-metal alloys, in fact, exhibit nanometer-scale chemical segregation, resulting in non-local angular momentum transfer during all-optical switching.^21^ Independent from material inhomogeneities, order recovery after optical excitation was also shown to involve the spatially inhomogeneous transient localization and coalescence of magnons, mediated by strong non-local spin currents.^22^ However, contrary to current-induced switching,^23^ the role of the boundary of the switched area has remained largely unexplored in HI-AOS. The present paper addresses this gap.

Boundary effects in HI-AOS are interesting because switching of an area that is smaller than the extent of the magnetic film—as is the case in most experiments—necessarily leads to the formation of a domain wall. At equilibrium, the magnetic order parameter $M=(M_{x},M_{y},M_{z})$ rotates continuously and with constant magnitude $M_{s}$ in such a domain wall, from the out-of-plane “up” domain [ $M=M_{s}(0,0,1)$] via an in-plane orientation [ $M=M_{s}(\text{cos}(\psi ),\;\text{sin}(\psi ),0)$] to the “down” domain [ $M=M_{s}(0,0,-1)$], with ψ defining the domain wall angle. As the in-plane component of the order parameter is zero before the switching event and finite afterward, the formation of such a domain wall can be considered a distinctive phase transition on its own, typically accompanied by spontaneous symmetry breaking due to degeneracies in the in-plane orientation ψ (i.e., the Bloch component of the domain wall)^24^ unless the material is strongly chiral. Fundamental aspects of this phase transition remain unexplored, including the order of the phase transition, where, when, and how fast such a domain wall forms, and how it transitions into its final state. These questions become especially interesting when considering the different times^18^—and locations^21,23^—at which the rare-earth and transition-metal sublattices reverse their out-of-plane magnetic order. In fact, this reversal proceeds via transient vanishing of the order parameter in the individual sublattices but not via global order parameter quenching (unless the laser fluence exceeds the threshold for laser-induced demagnetization). Given the difficulties of studying this ultrafast, nanometer-scale, stochastic phase transition directly in time-resolved experiments, defects in the final state become the most accessible experimental evidence to reconstruct the physics at play.

**FIG. 1.**
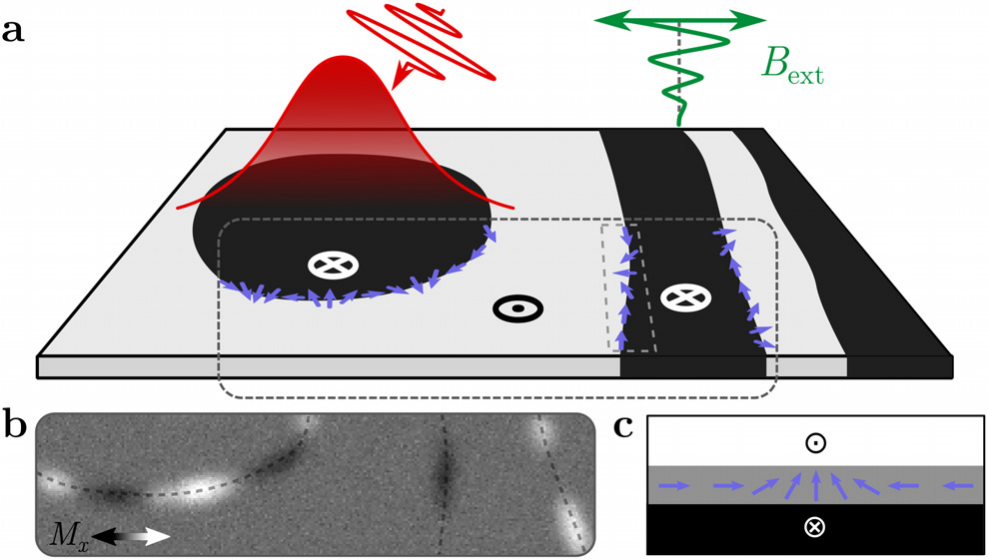
Schematic of the performed experiment. (a) The domain wall defect structure of optically nucleated bubble domains and stripe domains created by in-plane field cycling is investigated. (b) Expected XMCD x-ray absorption contrast along the domain walls for a tilted measurement geometry and difference imaging, corresponding to the magnetization configuration shown above. (c) Schematic top view of the magnetization structure of a single vertical Bloch line domain wall defect.

Here, we use x-ray vector spin imaging to investigate the defect density in magnetic domain walls in GdFe thin films after laser-induced all-optical switching and demagnetization as well as adiabatic field cycling [Fig. 1(a)]. In all experiments, we observe a high density of domain wall defects [Figs. 1(b) and 1(c)], which is remarkably robust against variations in the excitation. This is surprising because the pulse duration changes the speed of the transition (the time between switching of the transition metal and switching of the rare-earth element),^16,18^ which, by arguments of Kibble and Zurek, should result in scaling of the defect density with characteristic critical exponents.^10^ Based on evidence from time-resolved Kerr microscopy experiments, micromagnetic simulations, and comparison of materials with different pinning strengths, we suggest that this insensitivity to the actual switching process in our materials is due to the dominance of a second defect generation channel, namely, the post-nucleation motion of the domain wall over magnetic pinning sites.

## II. MATERIALS AND METHODS

### A. Materials

X-ray imaging experiments were conducted on Ta(3 nm)/GdFe(20 nm)/Pt(3 nm) and Ta(3 nm)/GdFe(20 nm)/Ta(3 nm) samples, sputter-grown with nominally identical GdFe alloy layers on x-ray transparent SiN-membranes. Both samples have perpendicular anisotropy and exhibit HI-AOS. The variation in capping materials, Ta and Pt, is motivated by the frequent use of these materials in spintronics applications; sizable spin–orbit interactions, such as interfacial anisotropy or Dzyaloshinskii–Moriya interaction (DMI), are not expected to emerge from these interfaces in the present case due to the large thickness of the magnetic film. This is confirmed later by the lack of a preferred chirality in the domain walls.

**FIG. 2.**
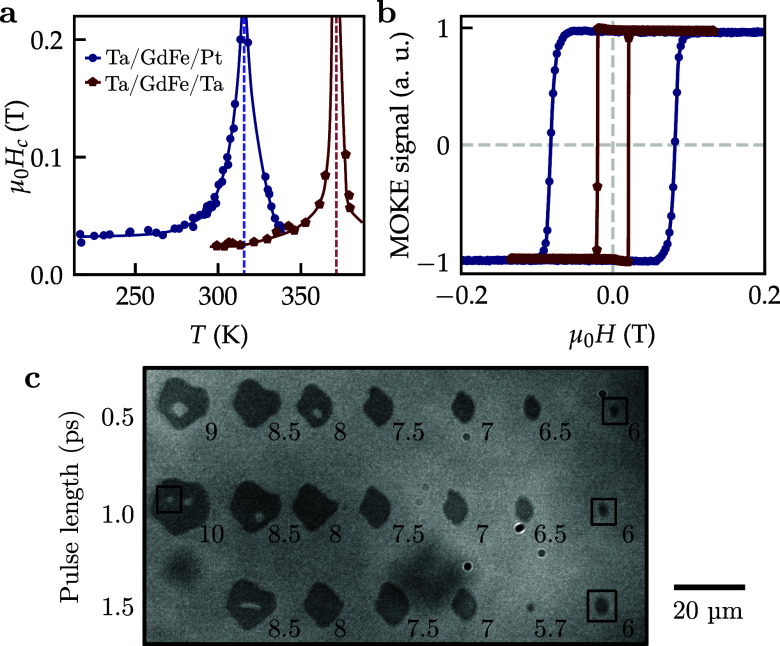
Magnetic material properties and preparations. (a) Coercive field vs temperature, acquired by zero-offset Hall-transport measurements. The estimated compensation temperatures are marked by dashed lines, and solid lines are guide to the eye. (b) Room temperature hysteresis curves recorded with MOKE. (c) Kerr microscopy image of laser-nucleated domains in Ta/GdFe/Pt. The laser pulse energy for each bubble is given in arb. units. Gray contrast corresponds to out-of-plane magnetization. Marked bubble domains were imaged with STXM.

Temperature-dependent measurements of the out-of-plane magnetic coercivity $H_{c}$, as shown in Fig. 2(a), yield the magnetization compensation temperature $T_{M}$ of the materials. Both materials compensate above room temperature, at slightly different temperatures of 323 K (Ta/GdFe/Pt) and 370 K (Ta/GdFe/Ta). We use the compensation temperature as a sensitive indicator of the actual composition of the magnetically active layer. Specifically, by interpolating composition-compensation-temperature graphs from literature,^25^ we reconstruct the compositions of the GdFe layers as Gd_0.255_Fe_0.745_ for the Ta/GdFe/Pt sample and Gd_0.259_Fe_0.741_ for the Ta/GdFe/Ta sample. The slight difference in composition is likely caused by diffusion of Gd into the Pt capping layer.^26^

The main difference between the two samples is in their magnetic pinning. Specifically, Ta/GdFe/Ta shows a sharp, square hysteresis loop at room temperature [Fig. 2(b)], indicating that switching at the coercive field $H_{c}=20\;\text{mT}$ is dominantly nucleation-limited. That is, the nucleation threshold of domains is above the pinning-threshold required to move domain walls. In contrast, Ta/GdFe/Pt shows a smoother field-induced transition and a higher coercivity of $H_{c}=82\;\text{mT}$. The more roundish shape of the hysteresis loop of Ta/GdFe/Pt indicates that domains nucleate at low fields but remain pinned until the field exceeds the depinning field. Notably, in either sample, the motion of domain walls takes place primarily at the coercive field, and for both samples, this driving field during field cycling is above the Walker breakdown field expected for materials with low DMI.^27^

### B. Preparations

Bubble domains were created with single shots of a linearly polarized, 1030 nm wavelength fiber laser in the initially uniformly out-of-plane magnetized film of both samples. Multiple laser fluences and temporal pulse widths above and below the AOS-threshold^19^ were chosen, such that all-optically switched domains of varying size and also demagnetized bubble domain clusters were created [Fig. 2(c)]. Very small switched domains below a sample-specific size threshold collapsed spontaneously within milliseconds to seconds after nucleation (as observed in similar setups^16^). The laser fluence was, therefore, increased such that domain sizes were above the collapse threshold. These nucleated domains were extraordinarily stable; no change in shape was observed between nucleation and x-ray imaging experiment. Even one year later, the domains still proliferated with their original shape.

We investigated the asymmetric Ta/GdFe/Pt sample also in a demagnetized state, achieved by cycling an in-plane field with decreasing amplitude, which resulted in stable, more than 100 *μ*m wide stripe domains.

### C. XMCD vector spin imaging

**FIG. 3.**
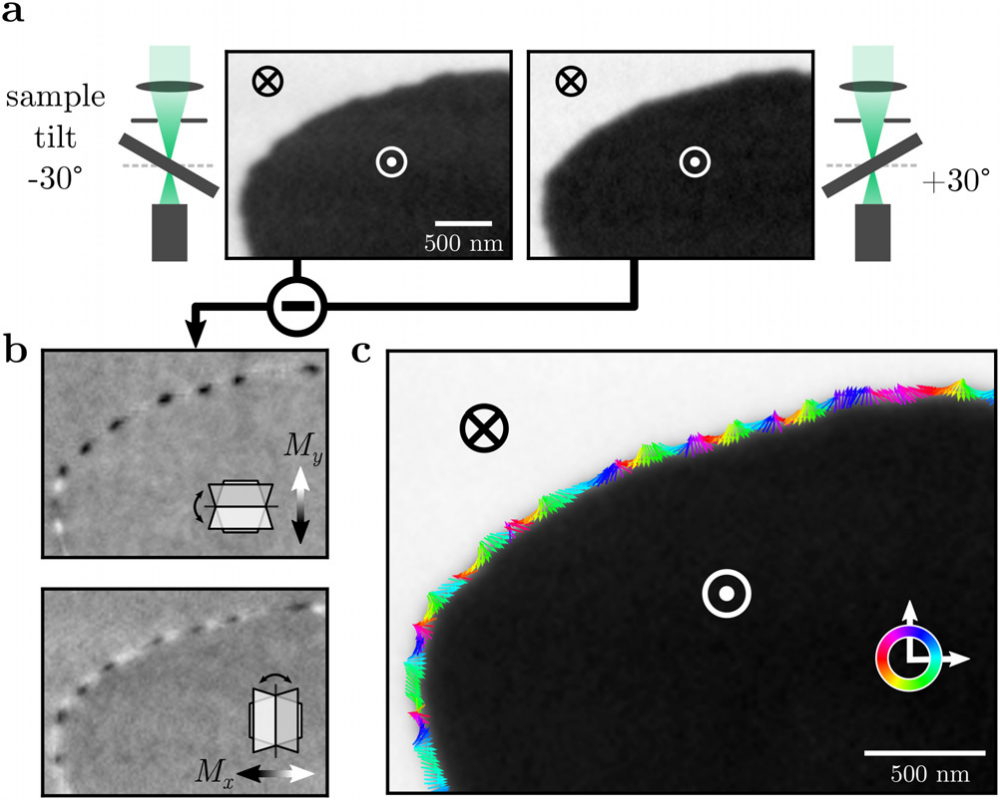
XMCD vector imaging and reconstruction. (a) STXM-images of a domain wall section, recorded with circular polarized x-rays for ±30° effective sample tilt. (b) In-plane components derived from the difference contrast of both tilts. Insets indicate the respective sample tilt directions. (c) Vector reconstruction of the domain wall magnetization shows rapid spatial rotation of the in-plane component.

Magnetic images were recorded by scanning transmission x-ray microscopy (STXM) at the MAXYMUS end station of the BESSY-II synchrotron^28^ with an instrument-limited spatial resolution of 25 nm (full width at half maximum, FWHM). Magnetic contrast was obtained using the x-ray circular magnetic dichroism (XMCD) at the Gd $M_{5}$ absorption edge (1184 eV). This method is sensitive to the component of Gd sublattice magnetization that is collinear to the x-ray beam. To gain vector spin information, we imaged the sample under various tilt and rotation angles, as illustrated in Fig. 3. Specifically, the samples were tilted by ±30° along the vertical axis (where the −30° tilt was realized by rotating the sample 180° around the out-of-plane axis, see Appendix) and further rotated by 90° around the out-of-plane axis. At each set of angles, we recorded images with sizable XMCD-contrast, see Fig. 3(a). The sums and differences of a set of effectively ±30° tilted images yield the components of the normalized magnetization m along the in-plane [*x*, *y*, comp. Fig. 3(b)] and out-of-plane (z) directions of the sample, respectively. By combining them all, we obtained full vector spin images [Fig. 3(c)]. In these full reconstructions, we find high densities of vertical Bloch line defects (points of chirality reversal, i.e., 180° rotations of the in-plane magnetization in the domain wall), but no generally preferred domain wall chirality. Note that this technique works particularly well for our materials since the domain wall width (measured to be roughly πΔ=60 nm, see Appendix) is larger than the achieved spatial resolution (44 ± 3) nm in our data (see Appendix).

In practice, we realized that vertical Bloch line defects are already visible in single in-plane-component images [Fig. 3(b)], where they manifest as black-to-white transitions on length scales where the domain wall itself is approximately straight. To speed up the experiment, we, therefore, omitted the 90° rotation in most cases and, henceforth, present images of a single in-plane component.

**FIG. 4.**
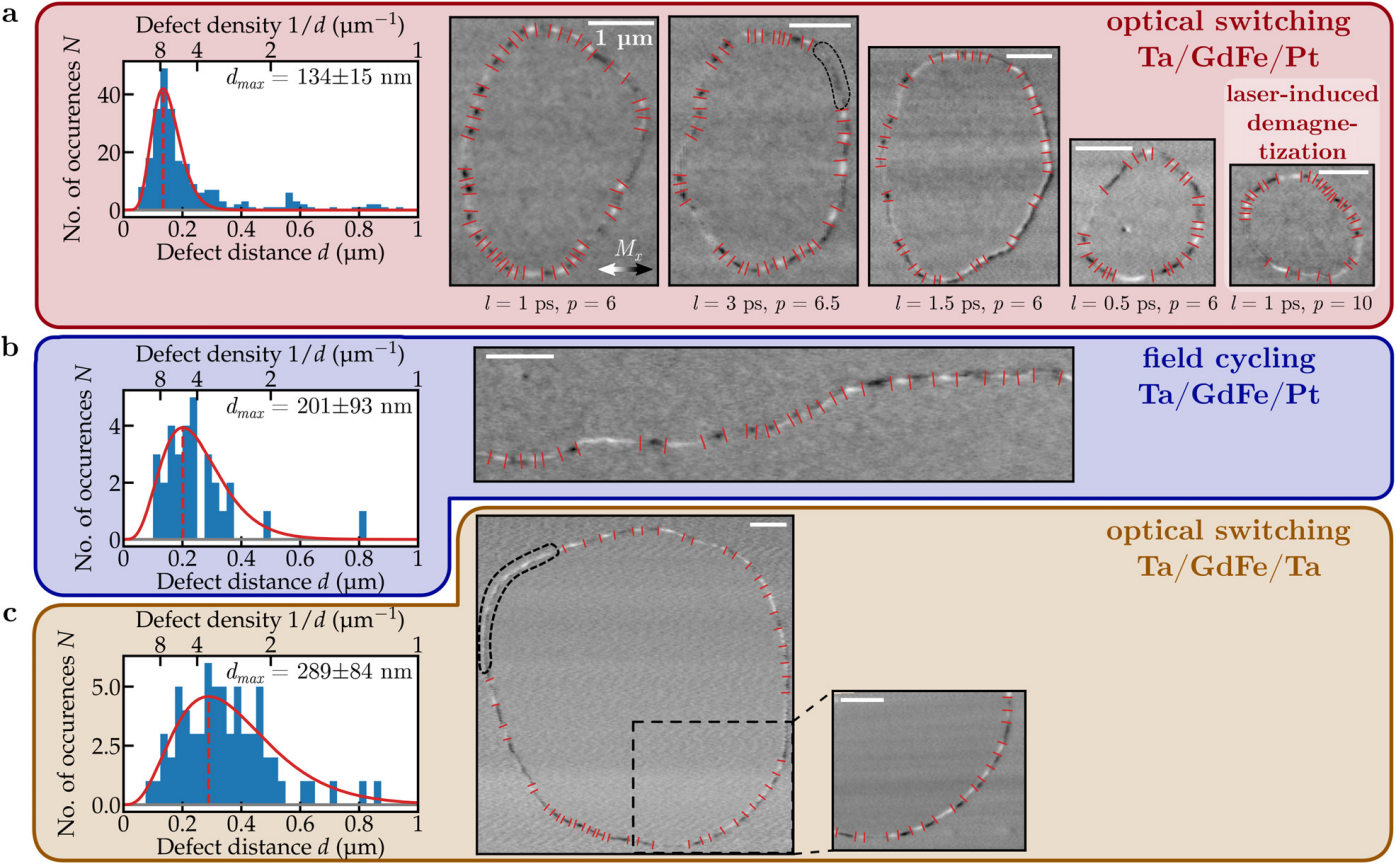
Domain wall defect density for various nucleation channels. (a) Distribution of distances between consecutive domain wall defects in optically nucleated domains in Ta/GdFe/Pt [in parts highlighted in Fig. 2(c)]. The histogram, the best fitting Γ-distribution, and the peak position with uncertainty are depicted as well as the original source microscopy-images, from which the data for the histogram were extracted. For each bubble domain, the pulse length *l* and energy *p* of the nucleating laser pulse are shown. (b) Image data and domain wall defect statistics of a domain wall created by field cycling in the same sample. (c) The same information for a bubble domain in Ta/GdFe/Ta created by AOS.

All raw images analyzed in our study are presented in Fig. 4. Some sections of the domain walls could not be reconstructed using our method and are marked with dashed lines in Fig. 4. These sections—comprising at most 20% of any imaged domain wall—either have a signal that is too weak to analyze or exhibit black-to-white contrast variations perpendicular to the wall, which do not align with the expected patterns illustrated in Fig. 1. The unusual contrast in these sections may originate from an exceptionally high density of vertical Bloch lines with separations below our imaging resolution. However, simulations indicate that this contrast could also stem from membrane wrinkling (see Fig. 9 in Appendix). Given the lack of conclusive data on defect density within these sections, we excluded them from our analysis. Note that this exclusion has minimal impact on our statistical analysis, as more than 86% of the total domain wall length exhibits clear black-white contrast modulations, enabling us to resolve the local in-plane magnetization of the domain walls and, consequently, the defects within them. Also note that for circular domains, this method overestimates the total number of vertical Bloch lines by two (see, e.g., Fig. 9 in Appendix). However, since we observe more than 28 vertical Bloch lines in each bubble domain, the impact of this effect is negligible for our study.

### D. Time-resolved Kerr microscopy

Time-resolved data of the all-optical switching process were recorded with wide-field Kerr microscopy. We used a Ta(3 nm)/Gd_22_Fe_72.2_Bi_5.8_(20 nm)/Ta(3 nm)-sample with added Bi for an enhanced Kerr-rotation angle.^29^ The setup consisted of a custom-built epi-illumination microscope^30^ with a 250 fs pulsed laser optical excitation at λ=1030 nm as well as the time-delayed probe illumination at λ/2=515 nm. Images were recorded at 1 kHz pump-probe repetition for probe delays between −15 ps and 1 ns. To reset the magnetic film between pump-probe events, a static magnetic field above $H_{c}$ was applied in the out-of-plane direction. For each delay, images with and without the excitation pulse were recorded and subtracted from one another. Contrast was normalized against the final state image, assuming that the nucleated domain is fully “up”-magnetized, while the material far away is “down”-magnetized. To remove high frequency image noise, a spatial Gaussian filter with standard deviation σ=200 nm was applied to each image.

### E. Micromagnetic simulations

We performed micromagnetic simulations to support our experimental observations to model how material inhomogeneities enable the creation of Bloch line defects during field-induced domain wall propagation. We used mumax3^31^ to simulate a 50 nm thick magnetic film with exchange stiffness $A_{\text{ex}}=7\;\text{pJ}/m^{3}$, saturation magnetization $M_{s}=198\;\text{kA}/m$, and perpendicular anisotropy $K_{u}=97.5\;{\text{kJ}/m}^{3}$, which were experimentally determined for a comparable DyCo-film of the same material family. The simulated area of 3000 × 3000 × 50 nm^3^ was spatially discretized into cells of size 3 × 3 × 50 nm^3^. For the Gilbert damping, we chose a generic value of α=0.5.

To simulate pinning within the material, the simulation area was divided into grains with an average size of 100 nm. Each grain was assigned a local anisotropy that randomly deviates from the mean in a Gaussian distribution of width $\sigma_{K_{u}}$. We modeled the pinning strength by varying $\sigma_{K_{u}}$ between 1 and 25 kJ/m^3^.

Each individual simulation for a specific set of pinning strength and driving field proceeded as follows. First, a single defect-free Bloch skyrmion was placed within the inhomogeneous material and relaxed to an initial stable state. Next, an out-of-plane field was applied that favors the magnetization direction within the skyrmion. The system was allowed to evolve for 15 ns or until the domain expanded to a volume of 25% of the simulation space. Finally, the magnetic field was removed again, and the relaxation dynamics were simulated until the system reached equilibrium.

## III. RESULTS

Our main observations are summarized in Fig. 4, where we show images of magnetic domain walls after all-optical switching, laser-induced demagnetization, and magnetic field cycling in our materials. All recorded XMCD difference images show strong in-plane contrast variations within the domain wall, corresponding to vertical Bloch line domain wall defects. Defects are present in both samples and all nucleation methods and parameters. We measured the distance between consecutive domain wall defects in each imaged domain wall. We find that the defect density is not constant along any domain wall. Instead, we observe sections with not only densely packed defects but also defect-free domain wall sections, seemingly without preferential direction.

We studied the defect density in bubbles created by various laser pulse lengths and intensities in Ta/GdFe/Pt. Surprisingly, we did not find any evident excitation parameter dependence (see Appendix). In consequence, we combined all of these datasets into a single histogram of the distances *d* between neighboring defects [Fig. 4(a)]. The distribution shows a prominent peak that can be well described by a Γ-distribution. The count of observed distances drops to zero at 50 nm, the realized imaging resolution in the experiment.

We use the fitted peak position as the measure for the characteristic defect distance and the reported fitting errors as the corresponding uncertainty. For optically created domains in Ta/GdFe/Pt, we obtain a characteristic defect-defect distance of $d_{\text{peak}}=(134\;\pm \;15)\;\text{nm}$ [Fig. 4(a)]. For a field-cycling-created wall in the same sample we extract $d_{\text{peak}}=(201\;\pm \;93)\;\text{nm}$ [Fig. 4(b)]. And for an all-optically created domain in Ta/GdFe/Ta we find $d_{\text{peak}}=(289\;\pm \;84)\;\text{nm}$ [Fig. 4(c)]. These peak distances are, even within their error bars, comfortably larger than the spatial resolution of the images. The same applies to the entire left-hand tail of the distributions and to the size of vertical Bloch lines in full vector spin images [Fig. 3(c)]. Therefore, we are confident that our statistical analysis is valid despite the finite resolution of our images.

**FIG. 5.**
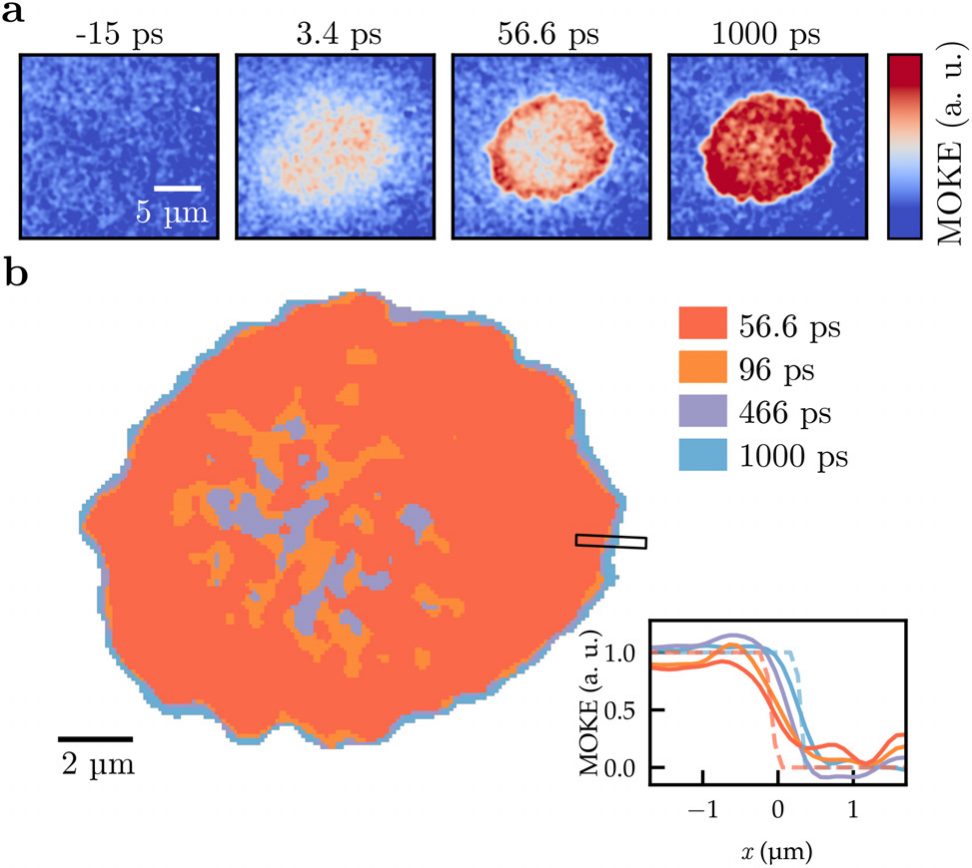
Time-resolved Kerr microscopy of all-optical domain nucleation. (a) Snapshots of the nucleation process before laser irradiation, during thermal demagnetization, and during the formation of the switched domain. The color scale is normalized such that the contrast at the largest delay corresponds to full negative (blue) and positive (red) magnetization. (b) Threshold of the MOKE-contrast for multiple states between the domain wall formation and the maximum recorded delay, which shows the gradual expansion of the domain outward. The inset shows one exemplary line-scan with the Gaussian-smoothed raw data (filled lines) and the corresponding profile after thresholding (dashed lines). The threshold corresponds to $M_{z}=0$, as determined in the fully demagnetized state.

We turn to time-resolved Kerr microscopy (Fig. 5) in search for the dynamics that could be responsible for the observed defect densities. Figure 5(a) shows multiple stages of the all-optical switching process. The initially homogeneously magnetized film is demagnetized shortly after the laser pulse hits the sample. After approximately 50 ps, the switched domain starts to form within the exposed area. The remagnetization of the material occurs first at the edges, where less heat is deposited by the laser pulse. Also, a fully connected domain wall around the switched area is first observable at this delay. The switching of the center of the domain follows subsequently. The apparent granular structure of the switched domain is experimental noise, which is also present in the data before the excitation [Fig. 5(a) at −15 ps]. Due to this graininess, we cannot clearly determine whether the domain wall forms initially as a circumference of the entire bubble or as small, local segments. What we clearly see, however, is a well-defined domain wall after 50 ps, which gradually expands outward. This domain wall motion continues up to the maximum measured delay time of 1 ns, as illustrated in Fig. 5(b). Note that expansion occurred even though the external resetting magnetic field was applied in the opposite direction.

The domain expands on average (290±90) nm between 50 ps and 1 ns. This value is considerable compared to the size of domains imaged in Fig. 4 and especially in comparison to the defect-defect distances and the domain wall width. The average domain wall velocity of 250 m s^−1^ during the expansion is safely above reported Walker breakdown speeds for comparable materials, even with finite DMI.^27^ Cause for the expansion could be the laser-induced transient thermal gradient. Notably, the domain wall moves away from the hot region, which is generally unexpected, but has been observed in ferrimagnets before.^32,33^

**FIG. 6.**
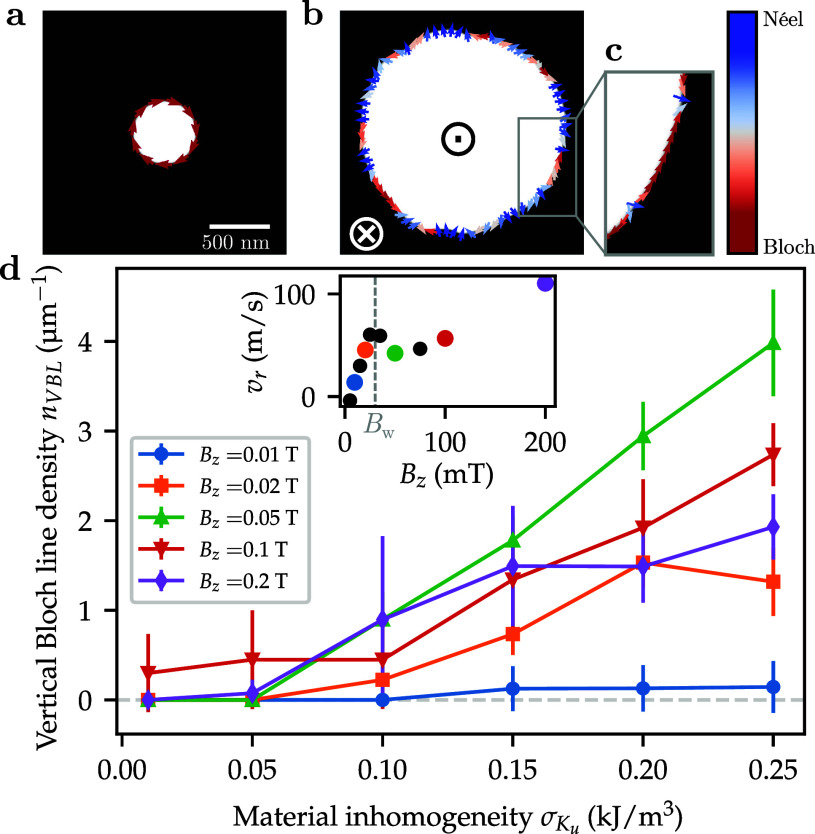
Micromagnetic simulations of field-induced bubble domain expansion in an inhomogeneous medium. (a) Initial homochiral Bloch domain and (b) final state bubble after field-induced expansion. (c) Bloch line defects after subsequent relaxation of the dynamic state. (d) Density of vertical Bloch line defects for increasing material inhomogeneity for various out-of-plane driving fields $B_{z}$. Each data point is the average of five simulations with randomized material inhomogeneity. The inset shows the dependence of the domain wall propagation speed on the applied driving field in a defect-free sample, with the determined Walker breakdown field.

**FIG. 9.**
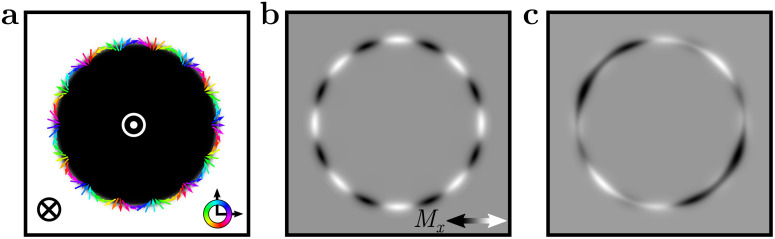
Impact of membrane wrinkling on the recovered in-plane information. (a) Magnetic bubble with high density of vertical Bloch lines. (b) Simulation of the XMCD-based ±30° difference image, assuming a perfectly flat sample. Note that 14 vertical Bloch line defects lead to a total of 16 contrast inversions along the domain wall. (c) Difference image for the same state with an additional permanent sample tilt of 2° around *x* and 7° around *y.*

Micromagnetic simulations provide a model to reproduce the expansion dynamics. Instead of a thermal excitation, an external magnetic field was used as a generic driving force for the expansion. We find that the originally defect-free domain wall accumulates defects during the expansion, as shown in Fig. 6(b). While some defects annihilate in the relaxation step, most of the Bloch lines still exist in the final state [Fig. 6(c)]. Generally, a higher material inhomogeneity leads to the generation of more vertical Bloch lines and increases their stability. Moreover, we find that driving fields around and above the Walker breakdown field $B_{W}$ result in a high density of defects, whereas fields far below the Walker breakdown create almost no domain wall defects. These observations are in line with previous theoretical studies that report on a critical field value above which Bloch lines nucleate during propagation.^34^ Generally, this value is at or slightly below the Walker breakdown field and—in the related case of horizontal Bloch lines, which can, themselves, cause the creation of vertical Bloch lines^35^—depends on the local anisotropy.^24^ The presence of areas with reduced perpendicular anisotropy, as in our simulation, therefore explains the Bloch line creation below the critical field value.^36^

## IV. DISCUSSION

Our findings suggest that the domain wall defect structure after HI-AOS is at least partially influenced by post-nucleation domain wall propagation dynamics. The speed at which domain walls propagate during this post-nucleation expansion is well above the Walker breakdown velocities. Even though the exact driving forces for the domain expansion after HI-AOS are not known, the likelihood of precessional motion at such velocities is high. The simulations indicate that under such precessional motion, domain walls accumulate vertical Bloch line defects if they move over sufficiently strong pinning sites. Intuitively, we can understand this behavior by considering that local variations of the magnetic properties lead to locally different domain wall precession frequencies. The consequence is a spatial mismatch in the internal spin structure. If the mismatch becomes too large, a pair of vertical Bloch lines forms.

The creation of defects during propagation does not exclude the possibility that some defects are already produced during the initial HI-AOS process. However, the experiments suggest that propagation-induced defects dominate in our experiments as this explains the insensitivity to the laser excitation parameters (e.g., pulse duration and fluence), the similarity of defect densities after HI-AOS and field cycling, and the larger defect density in the higher-pinning Ta/GdFe/Pt as compared to the lower-pinning Ta/GdFe/Ta.

The small increase in defects in optically created domain walls compared to field cycling provides a hint that some defects were present in the domain walls already at the moment of their creation. The increase is small, however, compared to the total number of present defects and lies within the uncertainty of the analysis. To properly investigate the nature of vertical Bloch line defects produced intrinsically by HI-AOS, the likelihood of defect production during post-switching dynamics must be strongly reduced. Our study suggests that the defect density in domain walls after magnetic field cycling can serve as a valuable metric in this optimization.

To conclude, our investigation has revealed a high density of domain wall defects within domain walls produced by helicity-independent all-optically switching. We attribute their origin to post-nucleation expansion dynamics, during which the nucleated domain walls move at high speeds over magnetic pinning sites. This interpretation is consistent with the insensitivity of the defect density on the stimulus that has triggered the switching, and the dependence on extrinsic pinning in the material. Our study demonstrates that systematic investigation of defects can provide insights into the dynamics of the switching process without requiring time-resolved experiments, which is particularly valuable for stochastic dynamics where stroboscopic imaging is impossible. The observed behavior contrasts with predictions based on the Kibble–Zurek mechanism, suggesting that in magnetic systems with strong pinning, post-nucleation domain wall motion dominates defect generation. More broadly, our findings illustrate how defect analysis can provide valuable perspectives on the limitations of speed, reproducibility, and stability in order parameter switching, with potential implications beyond magnetization to other switchable states, such as polarization in ferroelectrics or charge density in correlated electronic materials.

## Figures and Tables

**FIG. 7.**
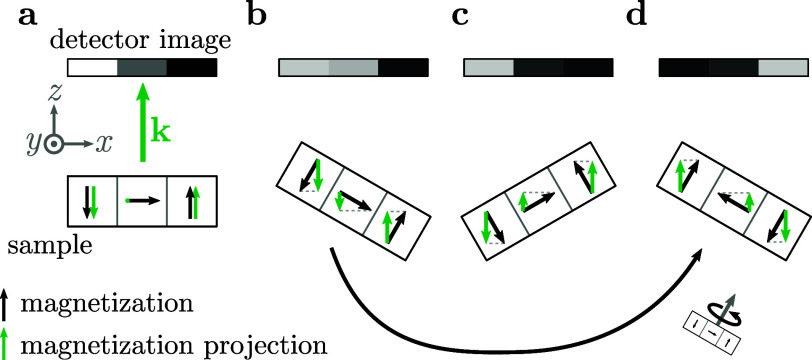
Sample manipulation steps performed during vector imaging. Shown is the cross-sectional view through a simplified Néel domain wall. Arrows show the local magnetization and the projection on k, which is proportional to the XMCD-contrast recorded by the detector. (a) Normal incidence, (b) +30° tilt, and (c) −30° tilt. (d) Additional rotation of the +30° tilted sample by 180° around the out-of-plane axis.

**FIG. 8.**
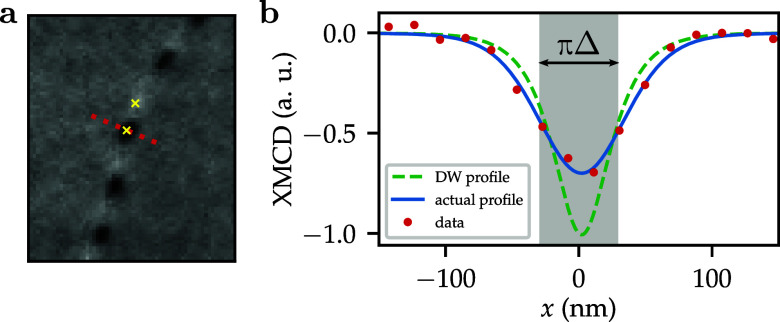
Estimation of imaging resolution and domain wall width. (a) ±30° XMCD difference image. Yellow crosses indicate sampling positions for the contrast measurement. (b) Domain wall cross section. Red dots show line-scan indicated in (a). Dashed line shows the isolated domain wall profile, while the continuous line shows the domain wall profile convoluted with the imaging resolution.

**FIG. 10.**
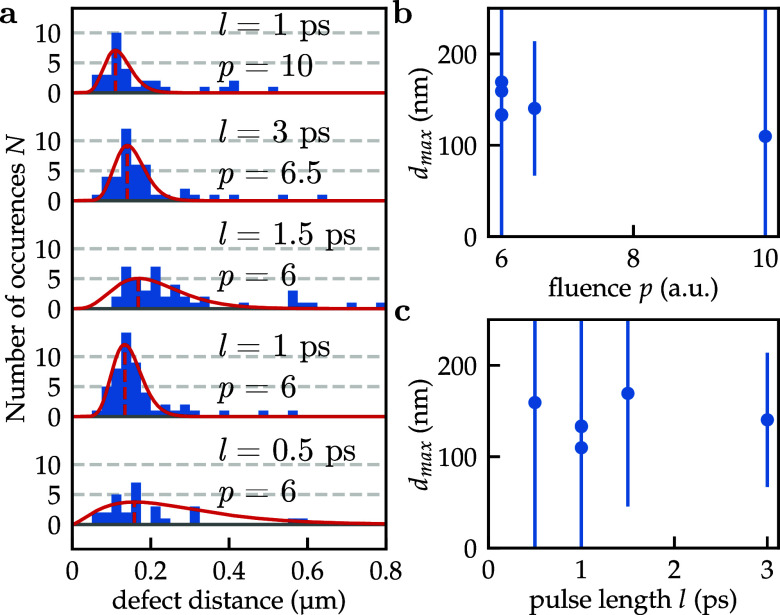
Dependence of the vertical Bloch line statistics on the laser parameters. (a) Histograms of the defect-distances and best fitting Γ-functions for each nucleated bubble in the Ta/GdFe/Pt sample with the annotated pulse length *l* and pulse energy *p* (in a.u.). (b) Fitted peak positions $d_{\text{max}}$ against the laser fluence. (c) Fitted peak positions against the pulse length.

## Data Availability

The data that support the findings of this study are openly available in Dataset 1 at https://doi.org/10.5281/zenodo.14499566.^37^

## APPENDIX: VECTOR SPIN IMAGING DETAILS

The experimental setup allowed for a sample tilt of +30° along the *y*-axis but not −30° along the same axis. XMCD absorption contrast is proportional to the projection M·k of the local magnetization M on the propagation direction of the x-ray beam k. Subtracting a set of ±30° images isolates the in-plane component because the projections of out-of-plane magnetic moments cancel out, while in-plane components perpendicular to the rotation axis add constructively [compare Figs. 7(b) and 7(c)]. Figure 7(d) illustrates that a sample rotation of 180° around the out-of-plane axis results in the same projected image contrast as −30°, albeit rotated.

We estimate the spatial resolution of the vector images from the contrast and the apparent width of domain walls in the in-plane images. Specifically, we know that some spins in a domain wall with vertical Bloch lines must point along the x-ray beam in the tilted geometry. Hence, we expect these sections to show sin(30°)/ cos(30°)≈57.7 % of the out-of-plane contrast within the domains (where the factor is due to the 30° tilt angle). Multiple measurements of the in-plane domain wall contrast, as indicated in Fig. 8(a), return values between 70% and 85% of this expected contrast amplitude. The in-plane domain wall magnetization profile follows $M_{\text{IP}}(x)=M_{s}/\text{cosh}(x/\Delta )$, with the domain wall width parameter Δ, from which the domain wall width can be estimated as πΔ [compare Fig. 8(b)]. The finite imaging resolution *r* results in an apparent smoothing of the domain wall profile, which is modeled by convolving with a Gaussian with FWHM *r*.

As shown in Fig. 8(b), the convolution of the physical domain wall profile with the Gaussian not only widens the observed profile but also reduces the amplitude, solely depending on the width of the Gaussian *r* compared to the domain wall width Δ. By adjusting the amplitude of the broadened domain wall profile to the measured values, we find ratios between $\frac{r}{\Delta }=1.6$ and 2.7. Notably, even the larger value for *r* is below the full domain wall width πΔ; hence, the resolution is small enough to reasonably resolve the domain walls and Bloch lines of similar size. Fitting a line-scan of the in-plane contrast with the convolved domain wall profile with the known ratio $\frac{r}{\Delta }$ results in a numeric estimate of Δ=(19±2) nm, which corresponds to a domain wall width of πΔ=(60±6) nm and a resolution of r=(44±3) nm. Note that the estimated resolution is weaker than the potentially attainable 25 nm of the MAXYMUS instrument and also spatially variable, due to the tilted sample geometry and resulting depth-of-field effects.

Simulations of the XMCD transmission contrast show that the in-plane contrast can also be affected by membrane wrinkling, which takes the form of an additional, inherent tilt of the magnetic film. The rotation of the two-dimensional magnetic pattern of a defect-rich bubble domain [Fig. 9(a)] was calculated by applying a set of consecutive rotations on the position and direction of each magnetic moment within the simulated magnetic layer. The XMCD-contrast was approximated as the local magnetization component collinear with the beam propagation direction. Figure 9(b) shows the difference of one simulated transmission image for a 30° rotation around the *y*-axis and one image for a 180° rotation around *z* followed by a 30° rotation around *y* [as described in Fig. 7(d)]. Figure 9(c) shows the difference image for the same transformations where an additional sample tilt of 2° around *x* and 7° around *y* was introduced ahead of the other rotations to imitate a wrinkling membrane. In both cases, the second image was rotated by 180° to restore the original orientation before subtraction. While the domain wall in Fig. 9(b) exhibits a clear black–white transition for each vertical Bloch line, the contrast is less clear in Fig. 9(c). Due to the additional tilt, the contrast modulations are suppressed, some sections are biased, while the domain wall contrast is reduced in others.

We note that we exclude scanning artifacts to be the origin of the black-and-white modulations in the in-plane difference images. Repeated scans of the same domain wall with a stable point-by-point scanning mode resulted in the same recovered in-plane component; hence, stochastic and systematic distortions did not impact the reconstruction. All small domains were imaged at this point-by-point scanning mode. Large domains were imaged in the faster line-at-once scanning mode, which has introduced distortion artifacts in the past. However, we found no signs of distortions in the present experiment, neither in direct comparison with point-by-point test scans nor in the process of combining images from different angles.

Multiple bubble domains resulting from various sets of laser excitation parameters were imaged in the Ta/GdFe/Pt sample. Figure 10 shows the distance statistics of domain wall defects for each parameter set. Within the fit uncertainties, no parameter dependence is apparent, neither for the pulse energy [Fig. 10(a)] nor for the pulse duration [Fig. 10(b)].
